# A Flanking Gene Problem Leads to the Discovery of a Gprc5b Splice Variant Predominantly Expressed in C57Bl/6J Mouse Brain and in Maturing Neurons

**DOI:** 10.1371/journal.pone.0010351

**Published:** 2010-04-26

**Authors:** Bethany H. Cool, Guy C-K. Chan, Lin Lee, Junko Oshima, George M. Martin, Qubai Hu

**Affiliations:** 1 Department of Pathology, University of Washington, Seattle, Washington, United States of America; 2 Department of Pharmacology, University of Washington, Seattle, Washington, United States of America; The University of Hong Kong, China

## Abstract

**Background:**

Gprc5b, a retinoic acid-inducible orphan G protein–coupled receptor (GPCR), is a member of the group C metabotropic glutamate receptor family proteins possibly involved in non-canonical Wnt signaling. Many GPCR transcripts are alternatively spliced, which diversifies this class of proteins in their cell- and tissue-specific signaling, regulatory and/or pharmacological properties. We previously generated *p97FE65* isoform-specific knockout mice that showed learning/memory deficits. In this study, we further characterized the *97FE65* null mice using cDNA microarray and RT-PCR analyses.

**Methodology/Principal Findings:**

We discovered a novel brain-specific C-terminal splice variant of Gprc5b, Gprc5b_v2, which was differentially expressed in *p97FE65* wild type and null mouse brains. The null mice were generated in 129/Sv ES cells, and backcrossed to C57Bl/6J for ten generations. We found that expression of Gprc5b_v2 mRNA in the brains of *p97FE65* null mice was dramatically down-regulated (more than 20 fold) compared to their wild type littermates. However, expression profiles of Gprc5b variants and SNP analysis surrounding the *FE65* locus suggest that the down-regulation is unlikely due to the altered FE65 function, but rather is caused by gene retention from the 129/Sv ES cells. Consistently, in contrast to ubiquitously expressed Gprc5b_v1, Gprc5b_v2 was predominantly expressed in the brain tissues of C57Bl/6J mice. The alternative splicing of the 3′ terminal exon also altered the protein coding sequences, giving rise to the characteristic C-termini. Levels of Gprc5b_v2 mRNA were increased during neuronal maturation, paralleling the expression of synaptic proteins. Overexpression of both Gprc5b variants stimulated neurite-like outgrowth in a neuroblastoma cell line.

**Conclusions/Significance:**

Our results suggest that Gprc5b-v2 may play a role during brain maturation and in matured brain, possibly through the regulation of neuronal morphology and protein-protein interaction. This study also highlights the fact that unexpected gene retention following repeated backcrosses can lead to important biological consequences.

## Introduction

G protein-coupled receptors (GPCRs), characterized by seven transmembrane domains, constitute important classes of evolutionarily conserved receptor proteins. They are also the most popular pharmaceutical targets due to their key roles in cell signaling [1]. Gprc5b, also known as retinoic acid inducible gene 2 (Raig2), is a member of the Raig subfamily of type 3 (family C) GPCRs. These proteins share homologies with the metabotropic glutamate receptors (mGluRs) in their seven transmembrane domain regions [2], [3]. In addition to the Raig and mGluR subfamilies, family C also includes GABA_B_, calcium-sensing, and pheromone receptors, all of which play significant roles in neuronal functions [4].

In addition to Gprc5b, three other Raigs have also been identified. In contrast to other family C members, the Raig receptors feature short amino termini more reminiscent of groups A and B of GPCRs [1], [3]. Although ligands have yet to be identified for any member of this orphan GPCR subfamily, recent evidence suggests that the Raig receptors may play a role in Wnt signaling. All of the four Raig receptors were found as potential Frizzled receptor-binding proteins to activate players in the non-canonical Wnt planar cell polarity pathway [5]. Overexpression of Gprc5b was also found to stimulate intracellular calcium release, possibly via activation of non-canonical Wnt calcium signaling [6], and to recapitulate non-canonical Wnt phenotypes observed with overexpressed Frizzled in Xenopus embryos [7]. Gprc5b is abundantly expressed in brain, with highest levels in the neocortex, hippocampus, and cerebellum [3], [8], although expression in peripheral tissues has also been observed [2], [3]. In brain, strong Gprc5b immunoreactivity was found in the cytoplasm of the cell body in pyramidal neurons, granule cells and Purkinje neurons; weak immunoreactivity was also detected in apical dendrites and neurites, and in astrocytes and oligodendrocytes [8].

FE65 is an adaptor protein that may influence the pathogenesis of Alzheimer Disease via its strong interaction with the intracellular tail of the beta-amyloid precursor protein (APP) [9]. FE65 may also play an important role in modulation of nuclear signaling [10], [11]. In searching for genes targeted by the FE65-APP pathway using cDNA microarray analysis, we discovered a novel C-terminal splice variant of Gprc5b, Gprc5b_v2, which was significantly down-regulated in brains of *p97FE65* (the full-length FE65) isoform-specific null mice, when compared with their wild type littermates. Further analyses revealed that the novel splice variant might be important for neuron/brain maturation. However, our evidence indicates that the differential splicing of Gprc5b was unlikely to have resulted from altered FE65 function, but instead was due to retained loci from 129/Sv ES cells that flank the *FE65* locus.

## Results

### Discovery of a novel, brain-enriched Gprc5b splice variant that is differentially expressed in *p97FE65* null vs. *p97FE65* wild type mice

Full-length FE65 (p97FE65) is a 97 kDa adaptor protein that has been shown to function as a transcriptional activator in the FE65/APP nuclear signaling pathway [10], [11]. In order to identify potential FE65 transcriptional targets, we performed cDNA microarray experiments on *p97FE65* null mice generated by our laboratory [12]. The *p97FE65* null clones were initially generated in 129/Sv-derived R1 ES cells and then the targeted clones were injected into C57Bl/6J blastocysts. At the time of microarray analysis, the mice had been back-crossed to C57Bl/6J mice for ten generations. We have previously shown that these mice exhibit deficits in learning and memory [12]. Gene expression profiles of the individual cerebral cortex of five littermate pairs of male *p97FE65* null and *p97FE65* wild type mice at six months of age were compared by microarray analysis with Affymetrix mouse 430A 2.0 chips (see Methods S1). Expression differences for all but one gene, Gprc5b (probe set 1451411_at), were less than 2-fold, with the false discovery rate predicted for all genes at 0.55>q>0.22 (Tables S1 and S2). The results are consistent with previous observations that gene expression differences in brain tissues measured by microarrays are usually small [13], [14], probably due to the cellular heterogeneity of brain tissues. Among the differentially expressed genes, 15 genes had greater than 1.4-fold differences in expression level (p<0.004), with 8 up-regulated, including Gprc5b (Table S1), and 7 down-regulated in *p97FE65* null mice (Table S2).

**Figure 1:**
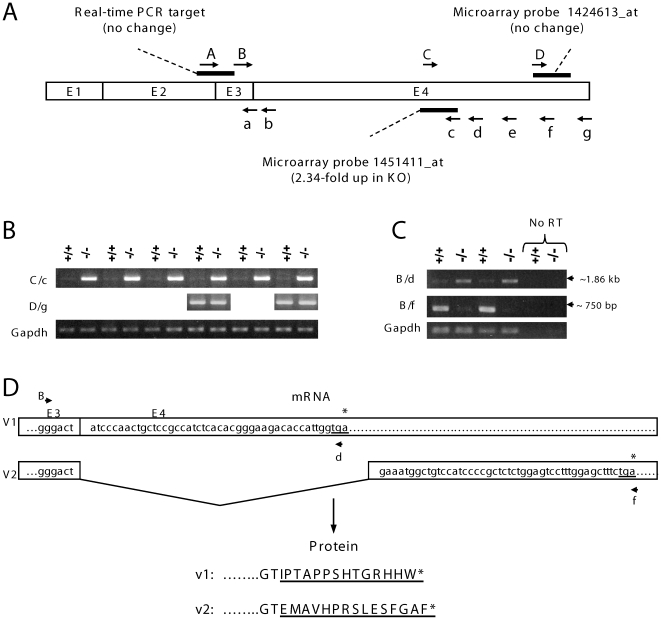
p97FE65 wild type and null mice express different Gprc5b splice variants in brain. **A**: Schematic drawing of Gprc5b transcript and location of primers used to amplify Gprc5b by RT-PCR. Forward and reverse primers are depicted by upper and lower case letters, respectively. Dark bars represent regions targeted by microarray probes and real-time PCR primer sets. **B–C**: RT-PCR analysis of Gprc5b transcripts present in forebrains of *p97FE65* wild type (+/+) and null (−/−) mice (3–12 months of age). Gapdh mRNA was amplified as control (bottom panels). B: Total mRNA was analyzed with Gprc5b primer sets C/c (upper panel) and D/g (middle panel). C: Total mRNA was further analyzed with primer sets B/d (upper panel) and B/f (middle panel). Primer set B/f amplified a band (∼750 bp) in wild type mouse forebrains, which was smaller than the size (∼2.7 kb) predicted from the Gprc5b mRNA reference (NM_022420). We were unable to detect the predicted product (∼2.7 kb) in either genotype using the PCR parameters described in MATERIALS AND METHODS, presumably due to difficulty in amplifying the large size of the amplicon. No RT (negative control): mRNA that was not reverse transcribed into cDNA. **D**: The cDNA and deduced protein sequences at the E3/E4 exon boundary for Gprc5b_v1, the predominant splice variant expressed in −/− brain (and also in 129/Sv brains; see Figures 3&4), and Gprc5b_v2, the predominant splice variant expressed in +/+ brain (and also in C56Bl/6J brains; see Figures 3&4). The protein sequences derived from the alternatively spliced E4 are underlined. The primers used in C are noted on the mRNA diagram as reference. Asterisks and underlined nucleotides denote stop codons.

Our further study was focused on Gprc5b because of its high differential expressions. Although real-time qPCR with pre-designed Assays-on-Demand primer/probe sets (Applied Biosystems, Foster City, CA) failed to confirm the differential expression of Gprc5b, we noticed that the sequences amplified by real-time qPCR did not overlap with those targeted by microarray probe sets (Fig. 1A), which could potentially have contributed to the discrepancy between microarray and qPCR results. We therefore employed semi-quantitative RT-PCR using primers that amplify the exact regions detected in microarray probe set 1451411_at (Fig. 1A). By this approach, we found a substantial up-regulation (up to 21.6- fold, determined by densitometry analysis) in Gprc5b in forebrains of *p97FE65* null mice compared to wild type mice, using primer sets (C/c and B/d) that are overlapped with microarray probe set 1451411_at (Fig. 1A) (p<0.00001; Figs. 1B–C, *upper panels*). In contrast, expression of Glyceraldehyde-3-phosphate dehydrogenase (Gapdh) was not changed (Fig. 1B, *bottom panel*); no differential expressions of Gprc5b in the same samples were detected when a downstream region was analyzed by either microarray (probe set 1424613_at) (Fig. 1A) or RT-PCR analysis (Fig. 1B, *middle panels*). The results indicated that (1) an alternative splicing event might occur in the 3′ region of the Gprc5b transcript; (2) the alternative splicing might differ between *p97FE65* null and wild type mouse brains; (3) the region targeted by microarray probe set 1451411_at and overlapped with primer set B/d (Fig. 1A) might be spliced out in *p97FE65* wild type mouse brains.

Further experiments confirmed the assumptions. RT-PCR analysis with primers B/f identified a PCR product (∼750 bp) in *p97FE65* wild type brains (Fig. 1C, *middle panel*); the fragment was barely detectable in *p97FE65* null mice. As the PCR product was considerably smaller than 2.7 kb, the expected size from the Gprc5b transcript reference (NM_022420), we reasoned that a 2 kb fragment in Gprc5b mRNA was spliced out in *p97FE65* wild type brains. Searching sequence databases, we found an EST sequence (CJ049931) from a cDNA library derived from C57Bl/6J mouse medulla oblongata that also suggested similar alternative splicing in this region. Both Gprc5b RT-PCR products (750 bp and 1.86 kb) depicted in Fig. 1C were purified and sequenced. The sequencing results confirmed that the RT-PCR products represented two splice variants of the Gprc5b gene, due to use of alternative 3′ acceptor sites for the terminal exon (exon 4). The variant predominantly expressed in *p97FE65* wild type brains contained a truncated exon 4, lacking approximately 2 kb of its 5′ portion compared to the reference (NM_022420). The other variant, abundantly expressed in *p97FE65* null mouse brains, was identical to the Gprc5b reference. In accordance with the system adopted by the Mouse and Human Genome Nomenclature Committees, we have chosen to name the Gprc5b transcript reference sequence (NM_022420) as Gprc5b_v1, and the novel splice variant, predominant in *p97FE65* wild type (i.e. C57Bl/6J) brain, as Gprc5b_v2. The alternative splicing also altered the protein coding sequences at the extreme C-termini, giving rise to the characteristic 14 amino acid residues in Gprc5b_v1, and 15 residues in Gprc5b_v2 (Fig. 1D; also see Discussion Section).

**Figure 2:**
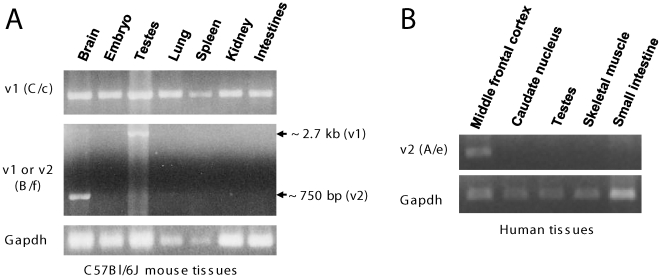
Expression of Gprc5b_v2 is enriched in brain. **A**. Total mRNA from C57Bl/6J mouse tissues (a 2-month old male) was amplified with Gprc5b primer sets C/c (upper panel) and B/f (middle panel) to detect Gprc5b_v1 and _v2. Primer set B/f can theoretically detect both _v1 and _v2. However, the Gprc5b_v1 product was rarely detected, due to its large size (∼2.7 kb). **B**. Total mRNA from human tissues (a 72 year old male) was amplified with Gprc5b primer set A/e to detect Gprc5b_v2. See Fig. 1A for relative positions of primers on the transcripts. Gapdh mRNA was amplified as control (bottom panels in A and B).

We examined expressions of the two splice variants in various tissues of C57Bl/6J mice. Interestingly, Gprc5b_v2, shown to have been predominantly expressed in C57Bl/6J brain, was not expressed (or expressed at below detectable levels) in other tissues (Fig. 2A, *middle panel*). Consistent with the previous evidence [2], [3], Gprc5b_v1 was detected in all examined tissues of C57Bl/6J mice, including brain, embryo, testes, lung, spleen, kidney, and intestine (Fig. 2A, *upper panel*). In addition, Gprc5b_v2 was also strongly expressed in human middle frontal cortex, but not in caudate nucleus and several peripheral tissues examined (testes, skeletal muscle, and small intestine) (Fig. 2B). These observations suggest that Gprc5b_v2 may play a special functional role(s) in brain.

### The splice variation found in *p97FE65* null mice is due to retention of a 129/Sv genomic sequence: the “flanking gene problem”

**Figure 3:**
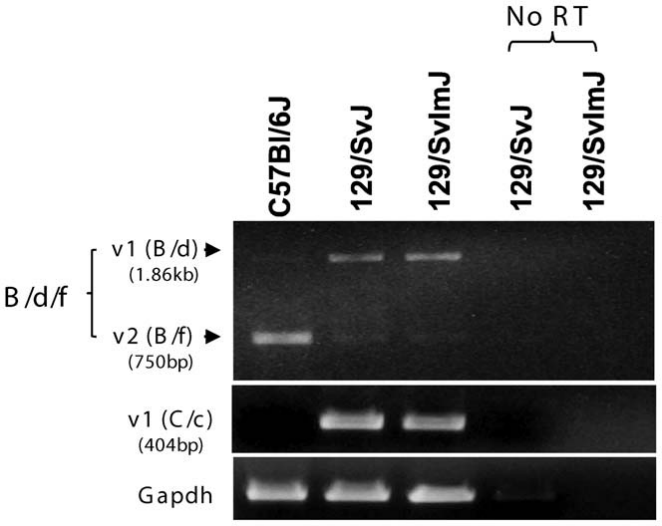
Mouse strain-specific expressions of Gprc5b splice variants. Total mRNA from C57Bl/6J, 129/SvJ, and 129/SvImJ forebrains was reverse transcribed, and then amplified with Gprc5b primer sets B/d/f (top panel), and C/c (middle panel) to detect Gprc5b_v1 and/or _v2. See Fig. 1A for relative positions of primers on the transcripts. Gapdh mRNA was amplified as control (bottom panel). No RT: mRNA that was not reverse transcribed into cDNA. Mice were approximately 2 months old when tissues were harvested for RNA isolation.

Interestingly, Gprc5b resides on mouse chromosome 7, where the *FE65* gene is located. The coincidence raised our concern that the differential expressions of Gprc5b splice variants in the brains of *p97FE65* null vs. wild type mice might not be due to altered FE65 function, but was rather, despite the extensive backcrossing to C57Bl/6J, due to the retention of genomic sequences flanking the *FE65* locus in strain 129/Sv mice (the strain of ES cells used to generate the knockout before backcrossing to C57Bl/6J) [12]. Expressions of the Gprc5b splice variants in C57Bl/6J and 129/Sv mice were therefore evaluated and compared. Strikingly, the brains of 129S1/SvImJ and 129X1/SvJ mice also predominantly expressed Gprc5b_v1, but not Gprc5b_v2 (Fig. 3). The results reinforced our hypothesis that retention of 129/Sv ES cell-derived genes flanking the *FE65* locus influenced the pattern of alternative splicing of *Gprc5b*. To further investigate this possibility, we searched for mouse single nucleotide polymorphisms (SNPs) in the Mouse Phenome Database (http://www.jax.org/phenome). We found unique SNPs in the *Gprc5b* gene that are differentially present between the 129/Sv (129X1 and 129S1), and C57Bl/6J strains. Genomic DNA sequencing across four SNP locations (SNP IDs: rs31179950, rs31329276, rs32286710, and rs31634095) within exon 4 of Gprc5b confirmed that the *Gprc5b* genomic sequence in *p97FE65* null mice matched strain 129/Sv but not that of C57Bl/6J. The results lead to the conclusion that the differential splicing of Gprc5b observed in brains of *p97FE65* null mice and their wild type littermates resulted from “the flanking gene problem” [15], and is not, therefore, indicative of an alteration of FE65 function in our memory-impaired mice bearing a partial knockout of *FE65*.

### Expression of Gprc5b_v2 is developmentally regulated in C57Bl/6J brain and cultured neurons

**Figure 4:**
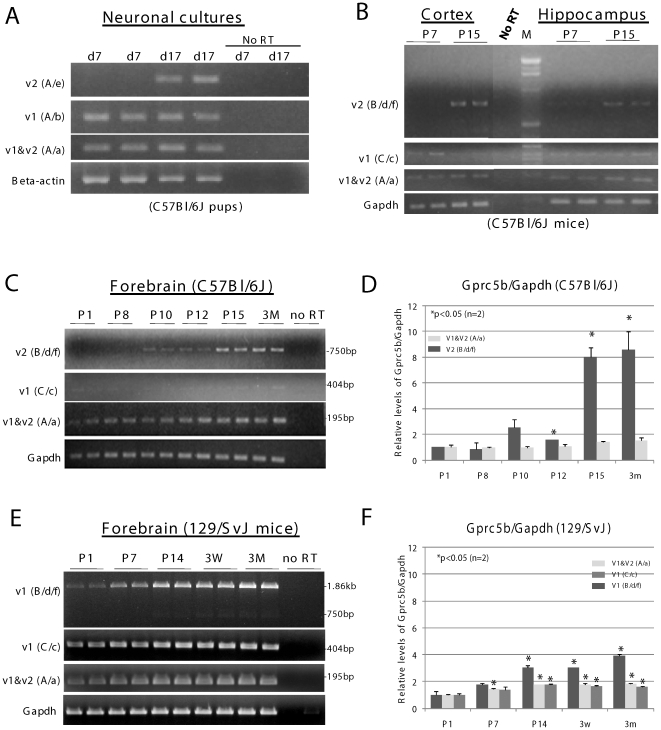
Expression of Gprc5b_v2 is increased in maturing neurons and during postnatal brain development. **A**. cDNA prepared from C57Bl/6J cortical neurons cultured for 7 days (d7) or 17 days (d17) was amplified with primer sets A/e, A/b, and A/a to detect Gprc5b_v2, Gprc5b_v1, and a region common to both splice variants, respectively. Cortical neurons were isolated from C57Bl/6J pups at postnatal day 0 (P0). Beta-actin mRNA was amplified as a control. **B**. Expressions of Gprc5b_v2, and Gprc5b_v1 in cortex and hippocampus of C57Bl/6J postnatal day 7 (P7) and day 15 (P15). Primer sets B/d/f, C/c, and A/a were used in RT-PCR to detect Gprc5b_v2, Gprc5b_v1, and a region common to both splice variants, respectively. **C**. Expressions of Gprc5b_v2, and Gprc5b_v1 in forebrains of C57Bl/6J mice during brain development. cDNA prepared from C57Bl/6J forebrains, was amplified with the same primer sets described in B. The tissues were collected from mouse pups at postnatal days 1 through 15 (P1–P15) and from 3 month-old (3M) adult mice. **D**. Quantitative analysis of expressions of Gprc5b splice variants detected in C. Relative intensities of Gprc5b_v2 (top panel of C), v1 & v2 combined (the 3^rd^ panel from top of C), and Gapdh (bottom panel of C) cDNA bands were determined by densitometry analysis with ScionImage software (www.scioncorp.com). Levels of Gprc5b were normalized to those of Gapdh. The two-tailed t-test was used for the statistical analysis (all comparisons were made to P1). Levels of Gprc5b_v1 (the 2^nd^ panel from top of C) were not quantified due to its low expression. **E**. Expressions of Gprc5b_v1, and Gprc5b_v2 in forebrains of 129/SvJ mice during brain development. cDNA prepared from 129/SvJ forebrains were amplified with the same primer sets described in B. The tissues were collected from mouse pups at postnatal days 1 through 14 (P1–P14) and at three weeks (3W), and from 3 month-old (3M) adult mice. **F**. Quantitative analysis of expressions of Gprc5b splice variants detected in E, as described in D. See Fig. 1A for relative positions of primers used. For B–F, Gapdh mRNA was amplified as a control. No RT: mRNA that was not reverse transcribed to make cDNA. M: DNA markers.

Expression of Gprc5b in neurons of the cortex, hippocampus and other brain regions [8] suggests that the alternative splicing event might be associated with some aspect of neuronal functioning. To test this hypothesis, we examined expressions of the two variants during primary neuron maturation *in vitro*, as well as in brain development *in vivo*. Semi-quantitative RT-PCR analysis showed that both C-terminal splice variants were expressed in cultured C57Bl/6J cortical neurons (Fig. 4A). However, expression of brain-specific Gprc5b_v2 increased with maturation of the primary neuronal cultures: Gprc5b_v2 levels in 17-day cultures were significantly greater (17 fold, determined by densitometry analysis) than those in 7-day cultures (p<0.05) after normalization to levels of beta-actin (Fig. 4A, *upper panel*). In contrast, Gprc5b_v1 levels remained relatively constant (p>0.05) (Fig. 4A, 2^nd^ *panel from top*). Overall Gprc5b levels, determined with a primer set (A/a) that amplifies a region common to both Gprc5b_v1 and v2, showed a trend towards an increase on day 17 (1.8 fold; p = 0.14) after normalization to beta-actin levels (Fig. 4A, *3^rd^ panel from top*). This probably resulted from the increased expression of Gprc5b_v2. Similar results were observed in repeated assays. The results indicate that expression of Gprc5b_v2 is regulated during the course of growth and differentiation of cultured neurons.

Consistently, we also observed up-regulated expressions of Gprc5b_v2 during development of C57Bl/6J brains (including forebrain, cerebral cortex, and hippocampus) from post-natal day 1 (P1) up to age 3 months (3M) (Fig. 4B–D). Levels of Gprc5b_v2 were barely detectable in brain tissues at P1 and/or P7/P8, but then increased at P10 and P12; high levels were detected at post-natal week 2 (P15). In forebrain, an 8-fold increase in Gprc5b_v2 levels was observed at post-natal week 2 (P15) (compared to P1) and the levels stayed relatively constant thereafter (Fig. 4C–D). In contrast, levels of Gprc5b_v1 remained relatively unchanged in these mice (Fig. 4B–C, *2^nd^ panels from the top*). Overall Gprc5b levels, including all known variants, showed an increased trend (Fig. 4B–C, *3^rd^ panels from the top*; Fig. 4D). The results indicate that expression of Gprc5b_v2 is up-regulated during development of brain. We also determined expressions of Gprc5b_v1 during development of 129/SvJ brains (we focused on forebrain only) from post-natal day 1 (P1) to the age of 3 months (3M) (Fig. 4E–F). In contrast to Gprc5b_v2 in C57Bl/6J, levels of Gprc5b_v1 in 129/SvJ were clearly detected at P1, and increased at P7. High and relatively constant levels were detected from P14 to 3M. Dependent upon which primer sets were used, magnitudes of the increase varied from 2 to 4 fold during 129/SvJ brain maturation (Fig. 4F) (vs. the 8-fold increase in Gprc5b_v2 levels during C57Bl/6J brain maturation shown in Fig. 4D). The results suggest that despite being ubiquitously expressed [2], [3], Gprc5b_v1 in 129/SvJ brain may partially complement the function of Gprc5b_v2 in C57Bl/6J brain. This interpretation is supported by observations with assays of neurite-like outgrowth in cultured cells, which indicate that both variants have the potential to enhance such outgrowth (see below).

### Gprc5b splice variants enhance neurite-like outgrowth

Recent evidence has shown that Gprc5b and its homolog, XRaig2, may play a role in non-canonical Wnt signaling [5], [16], a pathway known to impact processes involved in neuronal development and circuit formation, including dendritic arborization and neuronal polarity [17]. Therefore, we cloned the two mouse Gprc5b variants for assessment of their effects on neuronal cell morphology, starting with a human neuroblastoma cell line, SK-N-SH, as previously described [18], [19].

**Figure 5:**
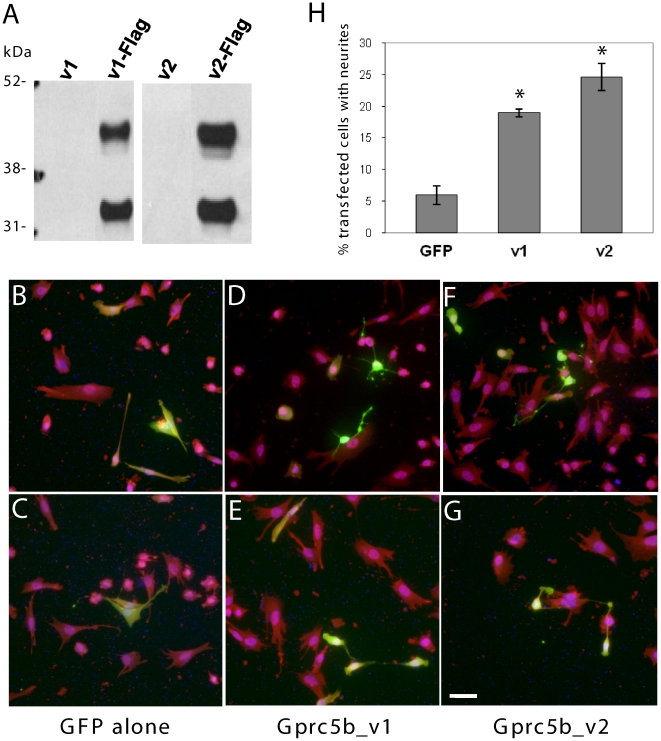
Gprc5b splice variants induce neurite-like outgrowth. **A**. Expression of Flag-tagged Gprc5b constructs. Human neuroblastoma (SK-N-SH) cells were transiently transfected with untagged or C-terminal flag-tagged Gprc5b_v1 or _v2 cDNA constructs and lysed 24 hours later. Western blotting with monoclonal Flag antibody reveals two protein bands running at ∼45 kD and ∼35 kD. **B–G**. Representative images of SK-N-SH cells expressing green fluorescent protein (GFP) alone (B, C), or untagged Gprc5b_v1 (D, E) or _v2 (F, G) in addition to GFP. At 24 hours post-transfection, cells were fixed for immunofluorescence analysis. Primary antibodies: GFP (green) and Gap43 (red); Hoescht DNA stain (blue). Images were collected at 20× magnification. Scale bar  = 50 micrometers. **H**. Quantitative analysis of percentages of Gprc5b-transfected cells bearing neurites. SK-N-SH cells were transfected with vector alone, or constructs coding for Gprc5b_v1 or _v2, as shown in B–G. A total of at least 342 GFP-positive cells from three independent transfection experiments per condition were counted and analyzed for the presence of neurite-like processes (which is defined as a process greater than or equal to one cell body in length). X^2^-test was used to analyze statistical significance (* = p<0.00001). The results show that overexpression of Gprc5b_v1 or _v2 increases the percentage of transfected cells (GFP-positive) bearing neurite-like processes by ∼3-fold or ∼4-fold, respectively, compared to the GFP control.

Gprc5b_v1 and _v2 were cloned into a bicistronic mRNA expression vector that was designed to express green fluorescent protein (GFP) separate from the gene of interest, via an internal ribosomal entry site (IRES) that follows the multiple cloning site [20]. GFP was utilized as a marker of transfected cells for morphological assessments. Flag tags were inserted at the C-termini to aide in expression analysis. Both tagged and untagged constructs were utilized for morphological studies to alleviate concerns that the tag might impact function. We noted two protein bands of about 35 and 45 kDa expressed from both of the tagged Gprc5b splice variants in transfected SK-N-SH cell cultures (Fig. 5A). Proteins of similar size were also expressed from C-terminal tagged XRaig2 mRNA injected into *xenopus* blastocysts [5]. The 35 kDa bands likely represent cleavage products, although it is also possible that they are a result of translation from alternative translation initiation sites. Further work is needed to confirm the putative cleavage event and to determine the biological importance of the extracellular N-termini.

Morphological assessment of SK-N-SH cells showed that overexpression of either untagged Gprc5b_v1 or _v2 induced an apparent increase in neurite-like processes in comparison to controls that expressed GFP alone (Fig. 5B–G). Similar results were also seen in cells overexpressing flag-tagged proteins (data not shown), suggesting that the tagging at the C-termini does not affect the function of Gprc5b to regulate cell morphology. Quantitative analysis confirmed visual impressions: overexpressions of Gprc5b_v1 and Gprc5b_v2 resulted in ∼3 fold (x^2^ = 23.18; p<0.00001) and ∼4-fold (x^2^ = 40.26; p<0.00001) increases in cells bearing neurite-like processes, respectively, in comparison to the cells overexpressing GFP only (a neurite-like structure is defined as a process equal to or greater than one cell body in length) (Fig. 5H). There was a trend of increased numbers of cells bearing neurite-like processes in Gprc5b_v2-transfected cells as compared to Gprc5b_v1-transfected cells (x^2^ = 3.00; 0.1>p>0.05). The results suggest that Gprc5b may play a role in regulation of cell morphology, such as neurite outgrowth. It remains to be determined whether Gprc5b_v2 plays a more potent role in modulating neurite outgrowth than Gprc5b_v1 in vivo, and whether brain-specific cofactors are required for fulfilling such functions.

**Figure 6:**
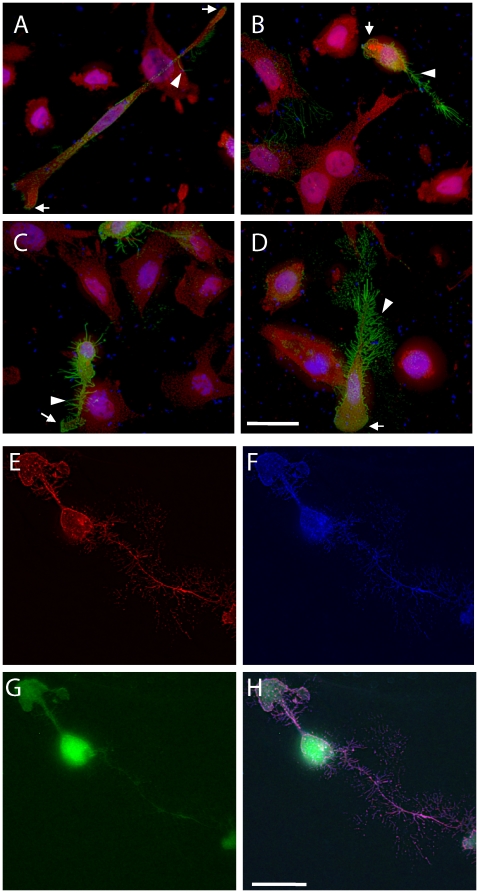
Colocalizations of Gprc5b with actin-rich membranes. **A–D**. SK-N-SH cells expressing flag-tagged Gprc5b_v1 (A, B) or Gprc5b_v2 (C, D) were fixed 24 hours post-transfection, and processed for immunofluorescence analysis. Flag (green); Gap43 (red); Hoescht DNA stain (blue). Note: Green denotes the localization of Flag-tagged Gprc5b, and not GFP, as 1) GFP is difficult to detect without primary antibody 24 hours post-transfection, 2) GFP localization is diffuse and does not concentrate at membranes (see Fig. 6G), and 3) use of far-red secondary to detect Flag reveals similar membrane localization (Fig. 6E). Arrows point to growth cones and lamelopodia; arrow heads point to filopodia. Images were collected at 40× magnification. Scale bar  = 50 micrometers. **E–H**. Representative SK-N-SH cell overexpressing Gprc5b_v2-flag-IRES-EGFP shows co-localizations of Flag immunoreactivity with actin-rich membranes. (E) Flag-tagged Gprc5b_v2 (red) is concentrated at membranes. (F) F-actin (blue) is also concentrated at membranes. (G) Conversely, the diffuse localization of GFP (green) is seen throughout the cell body. (H) Merged images of E–G. Images were collected at 100× magnification. Scale bar  = 25 micrometers.

The flag-tagged constructs were also utilized to assess the localization of the Gprc5b splice variants. Similar to human Gprc5b [3], both mouse Gprc5b_v1 and Gprc5b_v2 were transported to the plasma membrane in transfected SK-N-SH cells (Fig. 6A–D). They were present in dynamic actin-rich filopodia, lamelopodia, and growth cone membranes (Fig. 6E–H). The results are consistent with the notion that Gprc5b might regulate cytoskeletal morphology.

## Discussion

We have previously described the generation and characterization of isoform-specific *p97FE65* knockout mice that are unable to express the full-length 97 kDa FE65 protein, p97FE65 [12]. FE65 binds the intracellular tail of APP, domains of which appear to play key roles in the pathogenesis of Alzheimer Disease [21]. While the exact function of this brain-enriched multi-modular adaptor protein is unknown, several lines of evidence indicate that FE65 plays a role in modulation of APP metabolism, neurite outgrowth, neuronal positioning, and gene expression [9].

To generate knockout mice, an FE65 targeting vector was synthesized and transfected into 129/Sv-derived R1 embryonic stem (ES) cells, followed by injection of targeted ES cell clones into C57Bl/6J blastocysts. After the knockout line was established, behavioral tests were performed on F2 wild type, heterozygous, and knockout progeny derived from mating of F1 heterozygotes (129/Sv X C57Bl/6J). We found that *p97FE65* knockout and heterozygous mice exhibited poorer performances in comparison to *p97FE65* wild type mice in several behavioral tests that assess hippocampus-dependent learning and memory [12].

We further characterized the learning/memory-impaired *p97FE65* null mice after they had been back crossed with C57Bl/6J mice for ten generations. We used microarray analysis to determine the genes potentially targeted by FE65 and possibly involved in learning/memory. The results of the analysis led us to discover a novel Gprc5b splice variant whose expression was dramatically reduced in the knockouts. However, further studies revealed that the striking difference was due to the retention of 129/Sv ES cell-derived genes neighboring the *FE65* locus on mouse chromosome 7. This appears to be one of the few published examples of the “flanking gene problem” encountered in backcross experiments using transgenically altered 129/Sv ES cells. We show that at least one such 129/Sv-retained gene, Gprc5b, was differentially spliced in the brains of wild type vs. knockout animals due to strain differences in alternative splicing. Brains of *p97FE65* wild type (i.e. C57Bl/6J) mice were found to predominantly express a previously uncharacterized splice variant, Gprc5b_v2, while brains of *p97FE65* null mice as well as two 129/Sv substrains, the parental strains for the F1 hybrid R1 ES cells, were found to express a previously described variant, Gprc5b_v1. The latter is also enriched in peripheral tissues in both C57Bl/6J and 129/Sv mice. These results also suggest that in addition to FE65, Gprc5b, and possibly other retained 129/Sv genes on mouse chromosome 7 could also potentially contribute to the phenotypes observed in *p97FE65* null mice simply due to strain differences in alternative splicing and/or additional functions encoded within regional polymorphic genomic sequences. Although we are uncertain whether the retained genes around the *FE65* locus from 129/Sv strain had confounded the learning/memory deficits observed in *p97FE65* null mice [12], it is of interest that 129/SvEv, but not 129/SvJ mice, have been reported to be better learners than C57Bl/6J mice in the Morris water maze task [22]. The low expression of Gprc5b_v2 observed in at least two 129/Sv substrains, thus, may be less likely to have been related to the poor water maze performance found in the *p97FE65* null mice [12]. More importantly, our recent experiments using hippocampal infusions of *FE65* siRNA did in fact lead to defective learning in C57Bl/6 mice [23]. Those results support the hypothesis that FE65 itself plays a role in hippocampal-dependent learning and memory.

Only a few publications report on the “flanking gene problem” (e.g. [15], [24]). Many investigators, however, have chosen to simply ignore such caveats when formulating plans to create knockout and transgenic mice, either due to ignorance of the existence of recombinational “cold spots” or to assumptions that the risk that strain differences encoded within the flanking genes will impact the observed phenotypes is acceptably low. The presence of 129/Sv genomic sequence neighboring the *FE65* locus on chromosome 7 in *p97FE65* null mice is not unexpected if one considers the statistical distributions of homologous recombination. The *p97FE65* null mice used in our studies were backcrossed ten generations, reducing the representation of 129/Sv genes in comparison to F1 hybrid mice. However, on average, retention of 16 cM of DNA surrounding the target locus is expected even after backcrossing 12 generations [25]. Much longer flanking gene regions than this have been reported in some knockouts [26], probably reflecting variations in the frequencies of recombination along chromosomal domains. The Gprc5b gene is located ∼13.3 Mb (1 Mb = ∼1 cM) downstream of *FE65* on mouse chromosome 7. While we did not map the total region of retention in our mice, we did note 129/Sv-derived genomic sequences via SNP genotyping at the rs3673791 and rs3673847 locations within *Homer2* (a member of the family proteins that regulate group 1 mGluR function) ∼24 Mb upstream of the *FE65* locus. Mouse chromosome 7 is ∼137 Mb in length. Assuming continuous retention, more than 25% of mouse chromosome 7 in *p97FE65* null mice may represent the genome of 129-derived ES cells.

As Gprc5b mRNA and protein are abundantly expressed in the brain, particularly in neurons, it was of interest to compare the expression of C-terminal splice variants Gprc5b_v1 and Gprc5b_v2 in primary cortical neurons isolated from C57Bl/6J pups. We found that both variants were expressed. However, Gprc5b_v2 expression levels were elevated in more mature neurons, suggesting that the C-terminus of this variant may play an important role during neuronal maturation and/or in matured neurons. The time course of the developmental expression of Gprc5b_v2 is similar to that of proteins involved in the regulation of synaptic plasticity, including certain mGluR splice variants and NMDA receptor subunits [27], [28]. Based on the potential role in neurite outgrowth (Fig. 5H), the localization at membranes of active growth (growth cones, lamelopodia and filopodia) (Fig. 6), and the putative stimulatory effect on the actin-regulating Rho GTPases [5], we speculate that Gprc5b_v2 is up-regulated in mature neurons to impact the actin cytoskeleton in response to synaptic activity, possibly influencing dendritic spine morphogenesis and/or plasticity [29].

Alternative splicing of proteins within the brains of higher organisms is common, and may underlie the evolution of higher brain function [30] as well as certain neurological diseases [31]. Many GPCRs, including the mGluR subtypes that share homology with Gprc5b in their 7 transmembrane domains, are alternatively spliced [32]. mGluR C-terminal splice variants differ in signaling properties (but not G-protein coupling preferences) [33], mechanisms of internalization [34], and subcellular localization [35]. In general, GPCR isoforms differed at the C-termini may have distinct signaling and regulatory properties [32]. It remains to be determined how the unique C-termini of the Gprc5b_v1 and _v2 splice variants effect Gprc5b function in brain. However, based on the evidence that the expression time course of Gprc5b_v2 parallels the maturation of synapses, as discussed above, we propose that the unique extreme C-terminus of Gprc5b_v2 may code for a specific protein-protein interaction(s) with a ligand(s) or a domain(s) that couples with a macromolecular signaling complex involved in synaptic plasticity, such as the PDZ domain-rich signaling scaffolds found at the glutamatergic synapse [36], [37].

**Figure 7:**
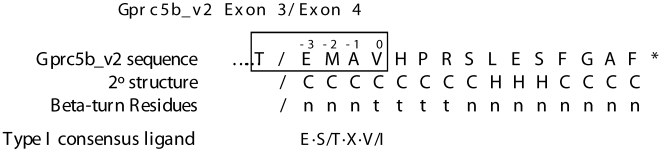
Gprc5b_v2 contains a putative internal type I PDZ ligand (T•E•M•A•V, boxed) in its unique C-terminus. The consensus ligand for type I PDZ domains is E·S/T·X·V/I (shown at the bottom). Secondary structure and beta-turn residues were predicted using the BetaTPred2 prediction program [44]. Secondary structure: C =  Coil; H =  Helix. Beta turn residues: t =  turn preferring; n =  not turn preferring.

Intriguingly, in search of unique sites of protein interaction within the novel Gprc5b_v2 C-terminus, we noted an internal sequence reminiscent of consensus Type I PDZ ligands (Fig. 7). Proteins containing PDZ domains act as scaffolds to hold together supra-molecular signaling complexes, and function in GPCR signaling to control signaling specificity, spatial organization of the glutamatergic synapse, and receptor trafficking/targeting [36], [37]. Type I PDZ domains interact with the consensus ligand E·S/T·X·V/I. However, divergent binding sequences have been described [38], [39]. Importantly, the putative ligand in Gprc5b_v2, T·E·M·A·V (Fig. 7), diverges from the consensus sequence at the -2 position. This spot is normally held by serine or threonine, which allow for phosphorylation-regulated binding [37]. However, we speculate that the threonine residue residing just upstream of glutamic acid (E) might adopt this role. While PDZ domains normally bind the last four or five amino acids at extreme C-termini, internal binding, often requiring the presence of a beta-finger turn immediately adjacent to the PDZ ligand, has been reported [40]. Importantly, a beta-finger turn is predicted immediately following the putative PDZ interaction sequence observed in the Gprc5b_v2 C-terminus (Fig. 7). Further proteomic studies are required to confirm the legitimacy of this putative ligand, and to identify the novel Gprc5b_v2 (and _v1) interacting proteins that steer its neuronal function.

In summary, we have identified a novel C-terminal splice variant of Gprc5b that is enriched in maturing neurons. Neuronal GPCRs are presently targeted to treat a variety of neurological disorders [32]. Additionally, it appears that both the brain-enriched splice variant, Gprc5b_v2, and the ubiquitously expressed splice variant, Gprc5b_v1, might regulate processes contributing to neurite-like outgrowth. While we recognize that much work is still needed to confirm Gprc5b function in neurite outgrowth, and to determine the underlying mechanism (e.g. the involvement in Wnt signaling), the present work is the first to present a potential function for Gprc5b in neuronal cells. Additionally, our discovery that Gprc5b is differentially spliced in the brains of *p97FE65* null mice in comparison to their wild type littermates due to the flanking gene problem lends further credence to the disclaimer that any delicate and functional phenotypes of transgenic and knockout mice that were generated using ES cells from a different strain than the breeding strain used to maintain the targeted mutation should be interpreted with caution. Even backcrossing ten generations will not solve the flanking gene problem.

## Materials and Methods

### Ethics Statement

All animal procedures were approved by the Institutional Animal Care and Use Committee at the University of Washington and the National Institute of Health Guide for the Care and Use of Laboratory animals.

### RNA isolation from tissues and cultured neurons

Various tissues were dissected from *p97FE65* wild-type and null littermates (3- to 12- months of age), from post-natal (days 1–15) and 2-month-old C57Bl/6J mice, and from post-natal (days 1–14), 3-week, 2 and 3-month-old 129X1/SvJ and/or 129S1/SvImJ mice. Brains of 129 sub-strains were purchased from The Jackson Laboratory (Bar Harbor, ME). Human brain (University of Washington Medical Center autopsy number 89-055) and peripheral tissues were previously described [41], [42]. Cortical neuron cultures were kindly provided by the laboratory of Dr. Daniel R. Storm, University of Washington, Seattle, WA. The neurons were isolated from the cerebral cortex of C57Bl/6J neonates and cultured essentially as previously described [43]. The following modifications were made to the culturing protocol: 1) Neurons were plated onto poly-D-lysine-coated 60 mm dishes at a density of 3×10^6^ cells per dish; 2) Neurobasal A media (Invitrogen, Carlsbad, CA) was used in place of Neurobasal media; 3) 2 mM GlutaMax (Invitrogen, Carlsbad, CA) was used in place of 0.5 mM glutamine in the culture media; and 4) no glutamate was added to the cultures at plating. Total RNA was isolated by homogenization of tissues or cultured cells in TRIzol (Invitrogen, Carlsbad, CA) or RLT lysis buffer (QIAGEN, Valencia, CA), according to the manufacturer's instructions. Mouse brain tissues and cells were further purified with an RNeasy mini kit (Qiagen, Valencia, CA).

### Semi-quantitative RT-PCR

Single-stranded cDNA (sscDNA) was synthesized from mRNA as previously described [41], except that M-MLV reverse transcriptase (RT) (Invitrogen, Carlsbad, CA) was used in place of SuperScript II. “No RT” controls (without addition of RT) were prepared in parallel. The sequence information used to generate Gprc5b primers (Fig. 1A) was obtained from Genbank accession number NM_022420. The forward primer sequences were 1) 5′-GCCTACATGGAGAACAAGGC-3′ (Gprc5b-A); 2) 5′-GGAGCAAAGATCTAGCAGCC-3′ (Gprc5b-B); 3) 5′-GCTGCTTTTGGAGGGTATGG-3′ (Gprc5b-C); and 4) 5′-CAAGCTGCGAGCACAACTTG-3′ (Gprc5b-D). The reverse primer sequences for Gprc5b were 1) 5′-ATTGAGCACGACGGCCATC-3′(Gprc5b-a); 2) 5′-GGGACTTCTCAGATCTCCTG-3′ (Gprc5b-b); 3) 5′-CTCAGCTCAGATGTACTGGC-3′ (Gprc5b-c); 4) 5′-TGAGGCATCTCAGGAGGTTG-3′ (Gprc5b-d); 5) 5′-AGCTCCAAAGGACTCCAGAG-3′ (Gprc5b-e); 6) 5′-GTCCCAATATGGTGGCATGC-3′ (Gprc5b-f); and 7) 5′-GCAAGTCAGCCAGGATAAGG-3′ (Gprc5b-g). The forward and reverse primer sequences for human Gapdh were 5′-CATCACCATCTTCCAGGAGCG-3′ and 5′-TCTCATGGTTCACACCCATGACGA-3′, respectively. The forward and reverse primer sequences for beta-Actin were 5′-TACAATGAGCTGCGTGTGGC-3′ and 5′-GCCAGAGCAGTAATCTCCTTCT-3′, respectively. PCR was performed using a Biolase DNA Polymerase kit (Bioline USA Inc., Randolph, MA), as previously described [41]. After initial denaturation at 94°C for 4 min, cDNA was amplified using the following cycling parameters: 94°C for 30 s; 65°C (all Gprc5b and Beta-Actin primer sets) or 60°C (Gapdh) for 40 s; 72°C for 30 s (Gprc5b primer sets A/a, A/b, A/e, C/c and GAPDH), 45 s (Gprc5b primer set D/g and Beta-Actin), or 2.5 min (Gprc5b primer sets B/d, B/f, and B/d/f). The PCR products were electrophoresed on agarose gels containing ethidium bromide to visualize the DNA bands.

### Plasmids

The pIRES2-EGFP vector (Clontech, Mountain View, CA) was kindly provided by Dr. Jane Sullivan, University of Washington, Seattle, WA, with the following modifications at its multiple cloning site: the BamHI site was removed and an EcoRV site was introduced by PCR-based site-directed mutagenesis. The original sequence was: gggatcc (BamHI  =  ggatcc); the modified sequence is: gatatcc (EcoRV  =  gatatc). Gprc5b sscDNA synthesized from mouse brain mRNA was amplified using forward (5′-AGTTCCTCGAGATGTTCCTGGTGTTAGAGAGAAAG-3′) and reverse (Gprc5b_v1: 5′-AATATGAATTCTCACCAATGGTGTCTTCCCGTG-3′; Gprc5b_v2: 5′-AATATGAATTCTCAGAAAGCTCCAAAGGACTCC-3′) primers containing *XhoI* and *EcoRI* restriction sites, respectively. The PCR products were then digested with *XhoI* and *EcoRI* restriction enzymes, and inserted at the same cloning sites of the pIRES-EGFP vector to generate Gprc5b_v1-IRES-EGFP and Gprc5b_v2-IRES-EGFP constructs. Flag-tagged constructs, Gprc5b_v1-flag-IRES-EGFP and Gprc5b_v2-flag-IRES-EGFP, were generated via site-directed mutagenesis with a QuikChange Site-Directed Mutagenesis Kit (Stratagene, La Jolla, CA). Two complementary oligonucleotides were used to insert the C-terminal Flag tags (Gprc5b_v1: 5′-CTCACACGGGAAGACACCATTGGGATTACAAGGATGACGACGATAAGTGAGAATTCTGCAGTCGACGG-3′ (sense) and 5′-CCGTCGACTGCAGAATTCTCACTTATCGTCGTCATCCTTGTAATCCCAATGGTGTCTTCCCGTGTGAG-3′ (antisense); Gprc5b_v2: 5′-CTCTGGAGTCCTTTGGAGCTTTCGATTACAAGGATGACGACGATAAGTGAGAATTCTGCAGTCGACGG-3′ (sense) and 5′-CCGTCGACTGCAGAATTCTCACTTATCGTCGTCATCCTTGTAATCGAAAGCTCCAAAGGACTCCAGAG-3′ (antisense)). Site-directed mutagenesis was initiated with the sense and antisense primers in separate reaction tubes. Each 20-uL reaction consisted of 300 ng plasmid DNA template, 20 pmol primer, 250 µM dNTP, and 2.5 units PfuUltra HF DNA polymerase with supplied reaction buffer (Stratagene, La Jolla, CA). After initial denaturation at 95°C for 2 min, the following cycling parameters were employed: 95°C for 1 min, 55°C for 1 min, 68°C for 12.5 min. Following 10 rounds of extension, sense and antisense reactions were combined into one tube, and run another 12 cycles. All inserts were verified by DNA sequencing.

### Transfection and Western blotting

SK-N-SH cell cultures were maintained as previously described [41]. Transfections were mediated by polyethylenimines (Polysciences, Inc., Warrington, PA) as described previously [11]. For each well of a 12-well plate, cells were incubated with premixed 1.2 µg DNA, 120 µl of Dulbecco's Modified Eagle Medium, and 6 µL polyethylenimines. Amounts of the transfection reagents were halved for cells seeded in 24-well plates. Cells were lysed in Laemmli buffer approximately 36–48 hours after transfection. Western blotting was performed with monoclonal anti-Flag M2 antibody (Sigma, St. Louis, MO) at 1∶2000 dilutions as described [11].

### Immunocytochemistry and quantititation of SK-N-SH cells bearing neurite-like processes

SK-N-SH cells grown on glass coverslips in 24-well plates were fixed about 24 hrs after transfection. Immunocytochemistry was performed essentially as described [11], except that cells were incubated with primary antibodies for 24 hrs at 4°C. Fluoromount G (Electron Microscopy Sciences, Hatfield, PA) was used to mount the coverslips on slides for microscopic analysis. Flag-tagged Gprc5b was analyzed with mouse monoclonal anti-Flag M2 antibody (Sigma, St. Louis, MO) at 1∶2000 dilution, GFP with an affinity purified rabbit polyclonal GFP antibody (Invitrogen, Carlsbad, CA) at 1∶2000 dilution, and a growth cone-associated protein, Gap43, with a chicken polyclonal antibody against Gap43 (Abcam, Cambridge, MA) at 1∶500 dilution. All secondary antibodies (Invitrogen, Carlsbad, CA) were used at 1∶400 dilutions, which included A488 (green) donkey anti-rabbit, A594 (red) goat anti-chicken, and A488 (green) goat anti-mouse. Following incubation with secondary antibodies, nuclei were visualized with the DNA-binding Hoescht dye (Invitrogen, Carlsbad, CA) at 1∶20,000 dilutions for 10 min at room temperature.

Inverted coverslips were systematically sampled for GFP-positive cells, starting at the upper left corner. Images (20x) were acquired using a DeltaVision deconvolution microscope (Applied Precision, Issaquah, WA), and processed using SoftWorx Explorer Suite (Applied Precision, Issaquah, WA) and Photoshop (Adobe Systems Incorporated, San Jose, CA). A total of at least 342 GFP-positive cells from three independent transfection experiments per condition were counted, and analyzed for the presence of neurite-like processes, which were defined as processes equal to or greater than one cell body in length.

### Statistical analysis

Data were expressed as mean ± SE. Statistical significance between any 2 groups was determined by the 2-tailed Student t-test or x^2^-test. P values less than 0.05 were considered significant.

### Database

Nucleic Acid Sequences for mouse Gprc5b_v2 (ACCESSION FJ529379) and for human Gprc5b_v2 (ACCESSION FJ529380) have been deposited in GenBank. All microarray data have been deposited in GEO, the Gene Expression Omnibus Database, under accession number GSE14274.

## Supporting Information

Methods S1 Microarray and statistics.
(0.04 MB DOC)

Table S1 Up-regulated genes in cerebral cortex of *p97FE65* null mice.
(0.05 MB DOC)

Table S2 Down-regulated genes in cerebral cortex of *p97FE65* null mice.
(0.05 MB DOC)
