# Supplementary material for: A Flanking Gene Problem Leads to the Discovery of a Gprc5b Splice Variant Predominantly Expressed in C57Bl/6J Mouse Brain and in Maturing Neurons
Source: PLoS One. 2010 Apr 26;5(4):e10351. doi: 10.1371/journal.pone.0010351 (PMC2859937; doi:10.1371/journal.pone.0010351)
Supplement: Methods S1 — Microarray and statistics. (0.04 MB DOC) [file pone.0010351.s001.doc]

SUPPLEMENTARY INFORMATION

METHODS

*Microarray and statistics* - All microarray data are MIAME compliant. Cerebral cortex tissues were dissected from five *p97FE65* wild-type and *p97FE65* null littermate pairs (6 months of age) for RNA purification and array analysis. RNA was converted into double strand cDNA with the SuperScript II double stranded cDNA synthesis kit (Invitrogen, Carlsbad, CA) according to the GeneChip^TM^ Expression Analysis Technical Manual (Chapter 1 section 2.1.15) (Affymetrix, Santa Clara, CA). Conversion of double strand cDNA into cRNA was accomplished by *in vitro* transcription using the BioArray High Yield RNA Transcription Labeling kit (Enzo Diagnostics, Farmingdale, NY) according to the manufacturer’s instructions. Fragmentation of purified cRNA for target preparation was done according to the protocol of the GeneChip^TM^ Expression Analysis Technical Manual (Chapter 1 section 2.1.22) (Affymetrix, Santa Clara, CA). Target hybridization was performed with Affymetrix MOE430A 2.0 oligonucleotide array chips at the University of Washington microarray facility according to the GeneChip^TM^ Expression Analysis Technical Manual.

Statistical analysis programs for selection of differentially expressed genes for further analysis were chosen with the assistance of a microarray core statistician (Dr. Richard Beyers) from the Nathan Shock Center at the University of Washington (Seattle, WA). The GC-RMA (Robust Multi Array) program (1) was employed to generate expression data that is normalized to probe-specific GC content in order to adjust for nonspecific hybridization signals. False discovery rates (q-values) and unadjusted p-values were calculated as previously described (2).

# SUPPLEMENTARY REFERENCES

1. Wu Z, Irizarry RA, Gentleman R, Martinez-Murillo F, and Spencer F. (2004) A Model-Based Background Adjustment for Oligonucleotide Expression Arrays. *Journal of the American Statistical Association* **99**, 909-917

2. Storey, J. D., and Tibshirani, R. (2003) Statistical significance for genomewide studies. *Proc Natl Acad Sci U S A* **100**, 9440-9445

3. Wang B, Hu Q, Hearn MG, Shimizu K, Ware CB, Liggitt DH, Jin LW, Cool BH, Storm DR, Martin GM (2004) Isoform-specific knockout of FE65 leads to impaired learning and memory. J Neurosci Res **75,** 12-24.
