# Supplementary material for: A Flanking Gene Problem Leads to the Discovery of a Gprc5b Splice Variant Predominantly Expressed in C57Bl/6J Mouse Brain and in Maturing Neurons
Source: PLoS One. 2010 Apr 26;5(4):e10351. doi: 10.1371/journal.pone.0010351 (PMC2859937; doi:10.1371/journal.pone.0010351)
Supplement: Table S1 — Up-regulated genes in cerebral cortex of p97FE65 null mice. (0.05 MB DOC) [file pone.0010351.s002.doc]

**Table S1: Upregulated genes in cerebral cortex of *p97FE65* null mice Table 1**: Upregulated genes in *p97FE65* null cerebral cortex.**Table 1**: Upregulated genes in *p97FE65* null cerebral cortex.**Table 1**: Upregulated genes in *p97FE65* null cerebral cortex.**Table 1**: Upregulated genes in *p97FE65* null cerebral cortex.

**(p < 0.004; q < 0.55)**

| Fold-Change | Gene Title | Gene Symbol | Chromosome † | Biological Process Description | Molecular Function Description | Cellular Component Description |
| --- | --- | --- | --- | --- | --- | --- |
| 2.35 | G protein-coupled receptor, family C, group 5, member B | Gprc5b | **7** (band F3, 111 Mb) | G-protein coupled receptor protein signaling pathway | G-protein coupled receptor activity metabotropic glutamate, GABA-B-like receptor activity | extracellular space membrane integral to membrane |
| 1.89 | IQ motif containing GTPase activating protein 1 | Iqgap1 | **7** (band D2, 73.1 Mb) | small GTPase mediated signal transduction; cell adhesion; cytoskeleton organization | Inhibits Cdc42 and Rac1 GTPase activity; calmodulin binding;  F-actin binding (*in vitro*) | Cytoplasm |
| 1.76 | thyroid hormone responsive SPOT14 homolog (Rattus) | Thrsp | **7** (band F1, 89.5 Mb) |  |  | Nucleus |
| 1.58 | PRP4 pre-mRNA processing factor 4 homolog B (yeast) | Prpf4b | 13 (band A4, 34.8 Mb) | nuclear mRNA splicing, via spliceosome mRNA processing; protein amino acid phosphorylation | serine/threonine and tyrosine kinase activity; ATP binding;  transferase activity | Nucleus  chromosome |
| 1.55 | A kinase (PRKA) anchor protein (yotiao) 9 | Akap9 | 5 (band A1, 4.0 Mb) |  | protein binding (PKA; PP1; InsP(3)R1; NR1 NMDA receptor subunit); kinase activity | pericentriolar material; cytoplasm |
| 1.5 | syntrophin, basic 2 | Sntb2 | 8 (band D2, 108.5 Mb) |  | actin binding calcium ion binding protein binding calmodulin binding | cytoskeleton membrane synapse |
| 1.43 | receptor (calcitonin) activity modifying protein 2 | Ramp2 | 11 (band D, 100.9 Mb) | intracellular protein transport; regulation of G-protein coupled receptor protein signaling pathway | receptor activity; protein transporter activity; coreceptor activity | extracellular space; integral to membrane |
| 1.4 | RIKEN cDNA 3100002M17 gene;  New name: Translocation protein 1 | 3100002M17Rik  (Tloc1) | 3 (band B, 31.7 Mb) | protein transport | protein transporter activity | integral to membrane |

† Mouse chromosomal information was obtained from project Ensemble (www.ensembl.org), a collaborator in the Mouse Genome Sequencing Consortium.
