# Supplementary material for: A Flanking Gene Problem Leads to the Discovery of a Gprc5b Splice Variant Predominantly Expressed in C57Bl/6J Mouse Brain and in Maturing Neurons
Source: PLoS One. 2010 Apr 26;5(4):e10351. doi: 10.1371/journal.pone.0010351 (PMC2859937; doi:10.1371/journal.pone.0010351)
Supplement: Table S2 — Down-regulated genes in cerebral cortex of p97FE65 null mice. (0.05 MB DOC) [file pone.0010351.s003.doc]

**Table S2: Downregulated genes**^¶^ **in cerebral cortex of *p97FE65* null mice**

**(p < 0.004; q < 0.55)**

| Fold-Change | Gene Title | Gene Symbol | Chromosome † | Biological Process Description | Molecular Function Description | Cellular Component Description |
| --- | --- | --- | --- | --- | --- | --- |
| 1.63 | myosin VIIa | Myo7a | **7** (band F1, 90.5 Mb) | cytoskeleton organization and biogenesis; perception of sound | motor activity actin binding protein binding calmodulin binding ATP binding | Cytoskeleton; myosin;  axon |
| 1.57 | Brain and kidney protein | Bk | **7** (band F3, 110.4 Mb) |  |  | Golgi trans face |
| 1.5 | RIKEN cDNA 0610006I08 gene | 0610006I08Rik | 19 (band B, 9.2 Mb) |  |  | Integral to membrane |
| 1.49 | ring finger protein 4 | Rnf4  (SNURF) | 5 (band B1, 32.9 Mb) | Inhibition of cell proliferation; regulation of transcription, DNA-dependent |  | nucleus |
| 1.44 | lectin, galactose binding, soluble 7 | Lgals7  (Galectin-7) | 3 (band F2, 93.1 Mb) | Inhibition of cell proliferation; apoptosis; heterophilic cell adhesion | sugar binding | nucleus |
| 1.44 | hepatoma-derived growth factor, related protein 3 | Hdgfrp3 | **7** (band D3, 74.1 Mb) | Cell proliferation |  | nucleus;  cytoplasm;  neurites |
| 1.43 | demethyl-Q 7 | Coq7 (Clk-1) | **7** (band F3, 110.6 Mb) | neurogenesis;  ubiquinone biosynthesis |  | Mitochondrion;  mRNA found in presynaptic terminals (squid photoreceptor neurons) |

^¶^ The *p97FE65* knockout transcript was not included in Table 2. This is because the two probe sets for FE65 (**1423892_AT** and **1423893_X_AT)** revealed only 1.08-fold (q=0.62) and 1.19-fold (q=0.49) down-regulation in *p97FE65* null mice, respectively, which did not meet the fold change cut-off parameter that we arbitrarily set at 1.4. We would like to point out that in *p97FE65* knockout mice, the targeting exon (exon 2) containing the 1^st^ translation initiation codon was deleted during RNA splicing. However, the FE65 transcript sequence, exon 1 as well as downstream of the skipped exon 2, was still expressed in the null mice (3). Probe sets **1423892_AT** and **1423893_X_AT** happened to be located at the 3’ end (between exons 13/14) of FE65, in a region common to both the wild type and knockout transcripts.

† Mouse chromosomal information was obtained from project Ensemble (www.ensembl.org), a collaborator in the Mouse Genome Sequencing Consortium.

**Table 2**: Downregulated genes in *p97FE65* null cerebral cortex.
